# The Structure-To-Function Relationships of Gammaherpesvirus-Encoded Long Non-Coding RNAs and Their Contributions to Viral Pathogenesis

**DOI:** 10.3390/ncrna4040024

**Published:** 2018-09-26

**Authors:** Gabriela Chavez-Calvillo, Sarah Martin, Chad Hamm, Joanna Sztuba-Solinska

**Affiliations:** Department of Biological Sciences, Auburn University, 120 W. Samford Ave, Rouse Life Sciences Building, Auburn, AL 36849, USA; gzc0025@auburn.edu (G.C.-C.); sem0036@tigermail.auburn.edu (S.M.); caw0038@auburn.edu (C.H.)

**Keywords:** gammaherpesviruses, long non-coding RNAs, RNA structure and function, viral pathogenesis

## Abstract

Advances in next-generation sequencing have facilitated the discovery of a multitude of long non-coding RNAs (lncRNAs) with pleiotropic functions in cellular processes, disease, and viral pathogenesis. It came as no surprise when viruses were also revealed to transcribe their own lncRNAs. Among them, gammaherpesviruses, one of the three subfamilies of the *Herpesviridae*, code their largest number. These structurally and functionally intricate non-coding (nc) transcripts modulate cellular and viral gene expression to maintain viral latency or prompt lytic reactivation. These lncRNAs allow for the virus to escape cytosolic surveillance, sequester, and re-localize essential cellular factors and modulate the cell cycle and proliferation. Some viral lncRNAs act as “messenger molecules”, transferring information about viral infection to neighboring cells. This broad range of lncRNA functions is achieved through lncRNA structure-mediated interactions with effector molecules of viral and host origin, including other RNAs, proteins and DNAs. In this review, we discuss examples of gammaherpesvirus-encoded lncRNAs, emphasize their unique structural attributes, and link them to viral life cycle, pathogenesis, and disease progression. We will address their potential as novel targets for drug discovery and propose future directions to explore lncRNA structure and function relationship.

## 1. Introduction: Defining Long Non-Coding RNAs

Only about 1.2% of the human genome encodes protein-coding genes, however a large majority is transcribed into non-coding RNAs (ncRNAs); products that seem to lack protein-coding capacity and are functional upon transcription [1,2]. This diverse group can be arbitrarily divided into: (i) small ncRNAs (sncRNA), transcripts shorter than 200 nucleotides (nts), which include microRNAs (miRNAs), small nucleolar RNAs (snoRNAs), piwi-interacting RNAs (piRNAs), and many others, and (ii) long ncRNAs (lncRNAs), transcripts longer than 200 nts [3]. This classification system is based solely on RNA length, and as such, does not reflect biological properties, biogenesis, stability, abundance, and/or mechanism of action.

The majority of lncRNAs are generated by RNA polymerase II, have a 5′ terminal methylguanosine cap, and are often spliced and polyadenylated [4]. Alternative pathways contribute to the generation of non-polyadenylated lncRNAs, which are likely expressed from RNA polymerase III promoters [5], and lncRNAs that are excised during splicing and small nucleolar RNA production [6]. No specific biochemical features can be exclusively ascribed to lncRNAs, but rather the lack of a defined open reading frame (ORF) suggests that many transcripts function intrinsically as lncRNAs [7]. Exceptions to these conventions include lncRNAs that have been shown to associate with polysomes and encode short or non-canonical peptides [8,9], and bifunctional mRNAs that are also lncRNAs [10].

Long ncRNAs have been identified as major players that are involved in the regulation of almost every stage of gene expression, the cell cycle, pluripotency, and modulation of host-pathogen interactions [3,11,12,13,14,15]. Despite wide distribution in genomes of complex organisms, only a small fraction of lncRNAs have been functionally and structurally characterized, and even less is known about virus-encoded lncRNAs [16,17]. In this review, we will highlight the most prominent examples of gammaherpesvirus-encoded lncRNAs, emphasize their multifunctionality in the viral life cycle and pathogenesis, and finally, propose a path for prospective studies.

## 2. The Role of lncRNAs in Gammaherpesviruses

Gammaherpesviruses form one of the three subfamilies of the *Herpesviridae*. They are characterized by their cellular tropism for lymphocytes and are distinct from alpha- and betaherpesviruses in molecular phylogenetic analyses [18]. Similar to host cells, herpesviruses produce lncRNAs and intriguingly, gammaherpesviruses encode the greatest number (Table 1). This viral lncRNA production allows for precise regulation of an unusual life cycle [11,13,14,19], which consists of two main phases: (i) latent, which is characterized by expression of only a few viral genes (latent genes) and no production of infectious virions; and, (ii) lytic, during which the virus expresses most of its genes, viral DNA is amplified, and progeny virions are assembled and released from the cells [20,21].

The gammaherpesviruses establish latency as a strategy for avoiding host immune surveillance and fusing symbiotically with the host for persistent lifetime infection [22]. However, the transition to the lytic phase of infection is critical for viral dissemination within and between hosts [23]. Timing of both phases must be finely tuned, and that daunting task can be achieved only by molecules that can either slip under the radar of host immune response and/or modulate cellular immune response. In most cases, lncRNAs appear to be more immune inert, which is perhaps due to a complex structure that sequesters cellular factors, preventing their detection by host surveillance system. In support of that notion, the Epstein-Barr virus (EBV)-encoded RNA1 (EBER1) interaction with the lupus antigen (La) has been shown to protect the 5′pppEBER1 from being recognized by cytoplasmic RNA sensors [24]. In addition, tampering with interferon signaling and cellular response genes establishes lncRNAs as essential modules of escape strategies used by viruses to avoid antiviral pathways. For example, Kaposi’s sarcoma-associated herpesvirus (KSHV) polyadenylated nuclear (PAN) RNA expression has been shown to interfere with the ability of transcription factors to activate the interleukin-4 (IL-4) promoter, and to knockdown the expression of RNase L, which is an essential interferon effector [25]. Further instances of extensive immunomodulatory lncRNA functions will be discussed in more detail throughout the manuscript.

## 3. Kaposi’s Sarcoma Herpesvirus (KSHV)-Encoded lncRNAs

**Figure 1:**
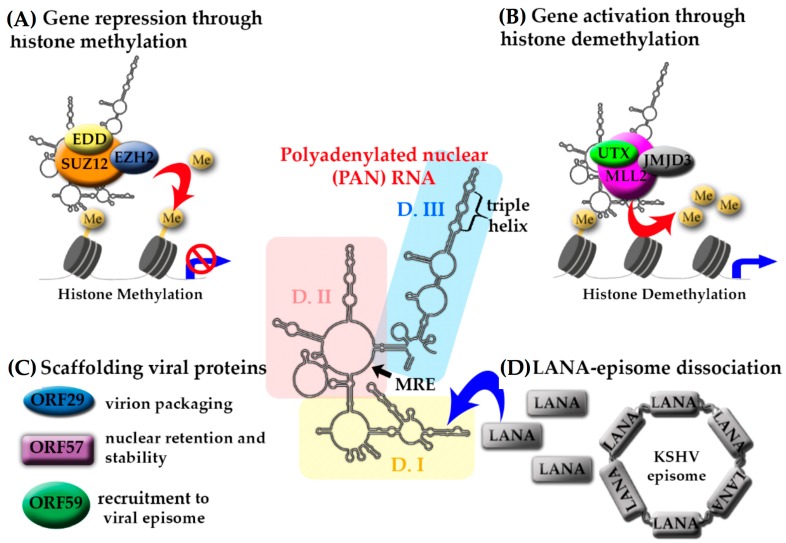
Structure-mediated multifunctionality of PAN RNA. The secondary structure of Kaposi’s sarcoma-associated herpesvirus polyadenylated nuclear RNA (KSHV PAN RNA) is represented in the middle with color-coded domains: I (yellow), II (pink), and III (light blue). The position of two cis-acting motifs that are involved in PAN RNA stability and functionality, Mta-responsive element (MRE) and triple helix, are indicated. (**A**) PAN RNA interaction with PRC2 components: EZH2 (blue), SUZ12 (orange), EDD (yellow), leads to histone methylation and gene repression; (**B**) The interaction of PAN RNA with UTX (lime green)/MLL2 (purple)/JMJD3 (light grey) targets histones for demethylation to increase gene expression; (**C**) PAN RNA interacts with viral proteins ORF29 (blue), ORF57 (purple), ORF59 (green) and LANA (grey); and, (**D**) The interaction of PAN RNA with LANA is partially responsible for the LANA-episome dissociation leading to KSHV lytic reactivation.

Kaposi’s sarcoma herpesvirus (KSHV) is the etiologic agent of Kaposi’s sarcoma, as well as certain B-cell lymphomas, including primary effusion lymphoma (PEL) and multicentric Castleman’s disease (MCD) [44,45,46]. KSHV encodes several lncRNAs [9,28,29,34,47] (Table 1), and PAN RNA is the most abundantly produced transcript during lytic reactivation [48,49]. PAN RNA has been shown to fold into three branched domains, each of which contains well-defined motifs connected by less structurally constrained regions (Figure 1) [50]. Domain I includes 9-nt element, termed the Mta-responsive element (MRE), which binds mRNA transcript accumulation protein (Mta, also ORF57), which modulates PAN RNA stability and function [51]. Domain III occludes a 79-nt long nuclear retention element (ENE) that sequesters the poly(A) tail of PAN RNA by formation of a triple helix [52]. This structural motif contributes to intracellular stability and allows PAN RNA to “escape” decay mechanisms. Domain II is characterized by a flexible conformation, which is likely to accommodate long-range tertiary interactions (e.g., formation of the ENE triple helix), support more compact folding in adjacent regions, and provide an accessible “landing pad” for protein interaction [50].

PAN RNA has been identified as a key player involved in regulation of almost every stage of viral gene expression, cell cycle, pluripotency, modulation of host-pathogens interactions, and the production of infectious virus [27,53,54]. It localizes mainly to the nucleus, yet, deep-sequencing studies also indicate its presence in the cytoplasm and in latently infected cells [55]. Arias and colleagues have suggested that the presence of PAN RNA in the cytoplasm might be explained by a potential protein coding capacity. They observed initiating ribosomes at the PAN start codon, elongating ribosomes throughout the body of the transcript, and the accumulation of releasing ribosomes at the stop codon [9].

PAN RNA knockdown experiments demonstrated compromised viral lytic gene expression and virion production [19,53,56], which is likely due to essential epigenetic regulatory roles. PAN actively participates in chromatin remodeling by recruiting the protein components of polycomb repressive complex 2 (PRC2) (Figure 1A), as well as the histone methyltransferase and the demethylases (Figure 1B) [53,55,57]. Using chromatin isolation by RNA purification (ChIRP-Seq), Rossetto et al. demonstrated the great extent to which PAN RNA manipulates viral and host gene expression programs [55]. Eighty-four cellular gene promoters that are involved in regulation of the inflammatory and antiviral responses (IFNγ, IL-18, IFNA16, and RNase L), cell death (TRIM68, RAD52, INPP5E, EPHB2, PAX2) and development (HIST1H4A, HIST3H3, PAX6, PAX5, CDKN2B), and thirty-five viral gene promoters involved in direct regulation of KSHV lytic gene expression (i.e., PAN, orLyt-L, K14, ORF4, ORF64, ORF50, ORF74), were shown to be directly recognized and regulated by PAN RNA [53,55]. PAN also interacts with the poly(A) binding protein C1 (PABPC1), which relocalizes to the nucleus during the lytic phase of KSHV infection [37,43]. This relocalization is directly caused by the shutoff exonuclease (SOX) protein, which downregulates the expression of host mRNAs and upregulates levels of PAN RNA [56]. Therefore, PAN RNA acts downstream of SOX, further contributing to viral manipulation of gene expression.

In addition, multiple viral proteins have been shown to associate with PAN RNA (Figure 1C). The interaction with ORF26 likely facilitates PAN packaging into virions [34,36], while the ORF59 likely facilitates the recruitment of PAN RNA to the viral episome [25,58]. PAN RNA has been also shown to regulate the function of the latency-associated nuclear antigen (LANA) protein (Figure 1D) [12]. During latency, LANA wraps around the KSHV episome and silences the expression of lytic genes. Lytic reactivation is marked by an abundance of PAN RNA, which sequesters LANA away from the episome, thereby relieving the repressive activity and facilitating the expression of lytic genes.

Other KSHV-encoded lncRNAs, including T1.2, T3.0, T6.1, and antisense-to-latency transcript (ALT) have been discovered, but only a few have been proven to be functional [31,32]. T1.5 is expressed from a region near one of the two origins of lytic replication (ori-Lyt). It is produced during the early stages of infection and is required for viral replication. T1.5 accumulates in the cytoplasm and is packaged into virions. T3.0 and T6.1 have the same transcription start site (TSS) and they are antisense to the replication and transcription activator (RTA/ORF50), but they do not inhibit RTA function. Although these three lncRNAs do not have canonical ORFs, all have been reported to be ribosome-associated, similar to PAN RNA [9]. ALT is a 10 Kb polyadenylated early lytic transcript expressed antisense to the major viral latency transcripts encoding LANA and the viral microRNAs [28]. In addition, ALT is on the same strand and is co-terminal with a bicistronic lytic transcript containing ORF K14 (v-OX2, which is a homolog of cellular surface receptor OX2) and ORF74 (vGPCR, viral G protein-coupled receptor) [26]. It has been suggested that the 3′ UTR that is common to ALT and the K14-ORF74 mRNA is likely regulated by microRNAs [28]. This overlapping arrangement of viral transcripts represents a strategy by which KSHV maximizes its coding capacity and level of gene regulation [28].

## 4. Epstein-Barr Virus-Encoded lncRNAs

Epstein-Barr virus, which is also known as human herpesvirus-4 (HHV4), is the causative agent of infectious mononucleosis [59] and it can lead to the development of Hodgkin’s lymphoma [60], endemic Burkitt’s lymphoma [59], and nasopharyngeal cancer [61]. This lymphotropic gammaherpesvirus undergoes five distinct phases of latency, and each can be characterized by the production of specific viral products, including lncRNAs. For example, EBV-encoded RNA1 and 2 (EBER1 and EBER2) are transcribed during all phases of latency, but EBV stable intronic-sequence RNA-2 (EBV-sisRNA-2) is detected only during latency III, while BHLF1 is expressed during latency I and III [30,31,62].

The EBERs are the most abundantly expressed nuclear ncRNAs in EBV-infected cells. More than 30 years after their discovery [30], they remain a functional puzzle to the study of EBV latency, mainly because gene deletion in EBER1/2-minus EBV bacterial artificial chromosomes shows no loss of viral latency establishment or tumorigenic potential [63]. When it comes to their classification, they fall in an arbitrary gap, as both EBERs are shorter than a typical lncRNA at ~180 nts each (Table 1). Yet, their biogenesis is distinct from that of miRNAs, prompting us to include them in the context of this review. EBERs are synthesized by RNA polymerase III [30] at approximately equal rates (Table 1), but they differ in half-lives, EBER1 at 8–9 h and EBER2 at 0.75 h [64]. While there is a low sequence homology between the EBERs (~54%), interestingly they share a highly conserved secondary structure [65].

**Figure 2:**
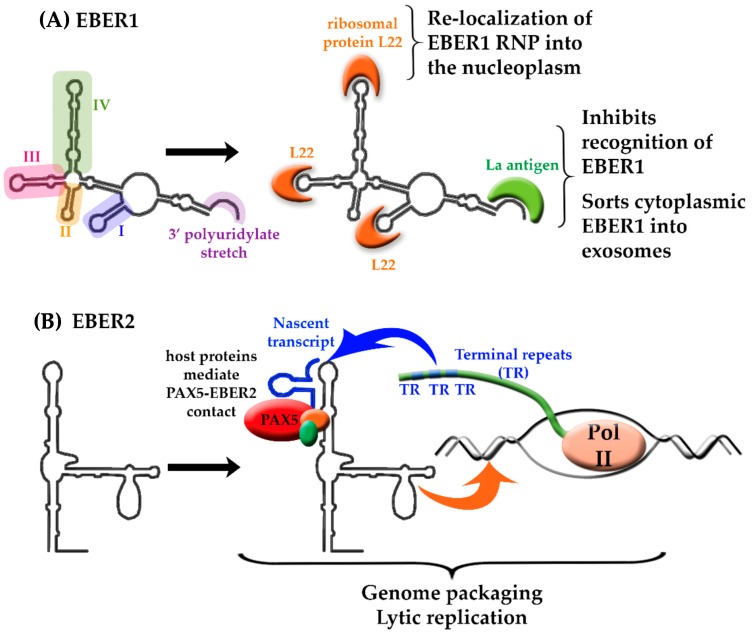
The structure-to-function relationship of EBER 1 and 2. (**A**) EBER 1 stem-loops I (violet), III (pink), and IV (green) create a scaffold for interaction with L22 (orange) resulting in the re-localization of EBER1:L22 into the nucleoplasm. EBER1 binds to the La protein (green) via the 3′ polyuridylate stretch (purple), shielding EBERs from recognition by host proteins; (**B**) EBER2 participates in the formation of a ternary complex with PAX5 (red), which involves host proteins (orange and green) and a nascent transcript (blue) expressed from the terminal repeats (TR) of the EBV genome. This interaction influences genome packaging and induces lytic gene expression, resulting in EBV reactivation.

The composition and functionality of all EBER ribonucleoprotein (RNP) complexes that are essential for EBV pathogenesis are as of yet undefined. It is known, however, that EBER1 folds into four stem-loops [32,65], and that stem-loops I, III, and IV each bind an L22 ribosomal protein (Figure 2A). L22 is normally present in the nucleoli and cytoplasm during latency, however, following EBV lytic activation, the complex relocates to the nucleoplasm [36,66]. Additional factors regulate the EBER1:L22 complex, including the binding of the interferon-inducible protein kinase R (PKR) and the association of La antigen with the EBER 3′ polyuridylate stretch [36]. The latter interaction has been proposed to dampen the recognition of a small fraction of cytoplasmic EBER1 by RNA sensors, and facilitate sorting of 5′pppEBER1 into exosomes in order to relay information about viral infection into neighboring cells [24]. In other words, EBER1 has been proposed to act as a stress-related signal, priming neighboring cells for 5′ppp-RNA detection [24]. In monocyte-derived dendritic cells, the exogenous delivery of EBER1 has been shown to induce the expression of interferon-related genes and inflammatory cytokine genes [67]. Equivalent process has been described in breast cancer stroma cells, which under stress, deliver endogenous 5′pppRNAs that are recognized by intracellular pattern recognition receptor (RIG-I) in tumor cells [68]. Thus, a conserved intercellular pathway involving EBER1 exists in which stress signals in the form of lncRNAs are transported between cells via exosomes [24]. Other known EBER1-mediated interactions reported include AU-rich element binding factor (AUF1)/heterogeneous nuclear ribonucleoprotein D (hnRNP D), a protein that is involved in destabilization of mRNA upon binding to AU-rich elements (AREs) [69]. The interplay between AUF1 and EBER1 potentially disrupts normal mRNA homeostasis and contributes to EBV-associated oncogenesis [70].

EBER2 has been shown to localize to the terminal repeats (TRs) of the latent EBV genome, likely to regulate EBV lytic reactivation (Figure 2B). Binding of the EBER2 stem-loop (nts 32–53) by both an RNA transcript expressed from TR locus and the B-cell transcription factor paired box protein 5 (PAX5) facilitates this localization. In addition, intermediary host proteins, including splicing factor proline and glutamine rich (SFPQ), non-POU domain-containing octamer-binding protein (NONO), and RNA binding motif protein 14 (RBM14), have been reported to mediate the PAX5-EBER2 interaction [71]. EBER2 and PAX5 have been proposed to act in concert, clearing adjacent chromatin of interfering factors to induce lytic gene expression. Disruption of the PAX5-EBER2 complex impacts genome packaging and the depletion of either PAX5 or EBER2 has been shown to decrease EBV lytic reactivation [66].

EBERs structural and functional similarity to the adenoviral ncRNAs VAI and VAII, suggests that, like VAI and VAII, the EBERs could interact with PKR. It has been confirmed in vitro that PKR dimerization, a requirement for activation, is inhibited in the presence of either EBERs or VAI/II [72]. Yet, in vivo studies utilizing multiple phosphorylation state-specific antibodies to monitor PKR activation within cells in response to interferon, challenge that hypothesis demonstrating that the EBERs are unable to inhibit phosphorylation of either cytoplasmic or nuclear PKR [73]. EBERs also regulate a variety of host cell genes, including those active in deamination, cell adhesion, apoptosis, and receptor signaling [74]. They have been shown to elicit transcription of cytokines, such as IL-9, IL-10, and IGF1 (insulin-like growth factor-1), which act as autocrine growth factors within EBV-infected cancer cells.

BHLF1, another EBV-encoded lncRNA, is 2.5 kb in length and it has an unusually high GC content (~78%) (Table 1). This lncRNA is transcribed from the LF3/BHLF1 promoter and it is the most abundantly expressed poly(A) viral transcript in chemically induced cells [14]. BHLF1 associates with one of the two origins of viral lytic replication to induce lytic reactivation, aiding initial strand separation and loading of core replication proteins [14]. Interestingly, around 10% of RNA-seq reads mapping to the BHLF1 locus contain G at position 40080 instead of an expected A residue encoded in the template, which is likely due to double-stranded RNA-specific adenosine deaminase (ADAR)-mediated deamination [37,75]. Because BHLF1 RNA actively influences the initiation of viral replication, ADAR-mediated RNA editing may also impact this process [35].

The long W repeat intron of EBV is responsible for expression of EBV-sisRNA-2, a structurally conserved, stable transcript. sisRNA-2 is produced during the most oncogenic phase of EBV latency (III), and folds into a thermodynamically stable 586 nt hairpin that contains intermittent bulges between canonically paired regions (Table 1) [33,76]. Similar thermodynamically stable hairpins have also been found in viral transcripts of other EBV strains, murine herpesvirus 4, and in the 3′ untranslated region (UTR) of some human transcripts [33]. Presently, the function of sisRNA-2 remains undefined, however, recent studies have shown that it is involved in interactions with human regulatory proteins, including: FUS, hnRNP L, hnRNP D/AUF1, HuR, hnRNP A1, LIN28, NONO, and hnRNP C [77]. These interactions may play roles in the emergence of EBV-associated diseases, which merits further investigation. Interestingly, EBER1 has also been shown to interact with hnRNP D/AUF1 [69], suggesting potential functional overlap.

Additional, lncRNAs bi-directionally transcribed from the origin of replication (oriP), during EBV, lytic reactivation have been described [11]. The leftward transcripts, oriPtLs, and the rightward transcripts, oriPtRs, are most likely non-coding, although part of oriPtRs includes the open reading frame for BCRF1/vIL10, viral analogues of human interleukin-10. OriPts associate with nuclear factors influencing the nuclear signaling environment, affecting lytic gene expression and viral DNA replication. Alike sisRNA-2, both of the transcripts are predicted to form large, evolutionarily conserved and thermodynamically stable hairpins that include family of repeat (FR) regions. The RNA-editing enzyme, ADAR is thought to bind both lncRNAs at the FR regions, leading to the extensive “hyperediting” of the hairpins [11]. Both oriPtL and oriPtR also interact with the multifunctional paraspeckle protein NONO, suggesting they comprise a part of the paraspeckle-based innate antiviral immune pathway. Interestingly, EBER2-PAX5 complex, as mentioned earlier in this section, has been also identified to associate with paraspeckles, in order to influence viral gene expression [78].

## 5. Murine Herpesvirus-68-Encoded lncRNAs

While murine herpesvirus-68 (MHV-68) is a natural pathogen of rodents, the pathogenesis of MHV-68 infection in mice mimics that of EBV/KSHV infection in humans with acute lytic viral replication, followed by the dissemination and establishment of persistent latency [79].

**Figure 3:**
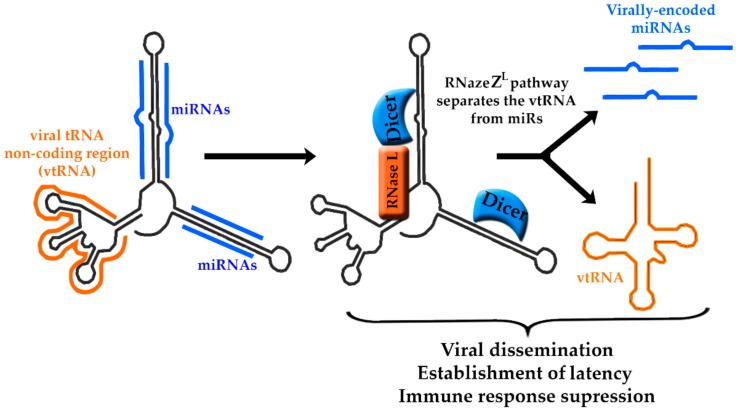
Maturation pathway of TMERs. Each TMER transcript contains a viral tRNA non-coding region (vtRNA, orange) and microRNA (miRNA) hairpins (blue). Through the RNaseZ^L^ pathway, the vtRNA is separated from the hairpins that are then processed by Dicer into miRNAs. TMERs are essential for the establishment of latency and viral dissemination, however, due to their close structural relationship their individual functions are not well defined.

During latency, MHV-68 produces eight tRNA-microRNA-encoded RNAs, referred to as TMERs, which are highly-abundant during asymptomatic infection and in proliferating B cells in the context of lymphoproliferative disease [80]. TMERs include a viral tRNA non-coding region, referred to as vtRNA, followed by two to four miRNAs sequences localized downstream of the vtRNA (Figure 3). Because of their tight structural arrangement, it has been a challenging task to separate the potential functions of the vtRNAs and miRNAs from each other [81].

When it comes to biogenesis of TMERs, the transcription utilizes the second type of RNA polymerase III (pol III) promotor system, comprised of three overlapping A box elements (TRGYNNARNNG) and a B box (RGTTCRANTCC), which are separated from one another by ~30–60 nts [82]. The promoter sequences recruit transcription factors, while simultaneously containing the D- and T-loops sequences that make up the vtRNA. The A and B boxes are required for the structural development of the pseudo-tRNA, which, in turn, facilitates the production of miRNA-producing stem-loops. Elimination of any of the box elements inhibits miRNA production. The tRNA and the miRNA portions of TMERs are co-transcribed and subsequently cleaved into separate components via the tRNA maturation pathway, referred to as RNaseZ^L^. On the other hand, the miRNAs are processed through an association with Dicer, and they are subsequently involved in the regulation of viral gene expression [38]. It has been proposed that the pol III transcription of tRNA stem-loops could also result in production of small interfering RNAs (siRNAs) through the RNaseZ^L^ pathway [83] (Figure 3).

The in vivo contribution of TMERs to MHV-68 biology has been established based by a panel of individual TMER mutant viruses [75,78]. It has been shown that most TMER mutants had little to no influence over viral latency, with the exception of TMER4, which has been established as a key mediator in MHV-68 hematogenous dissemination and latency. Interestingly, TMER4 vtRNA4 stem-loops, but not miRNAs, were shown to be essential for wild-type TMER4 activity, as they likely act as structural elements for protein binding that dictate RNA processing and TMER4 localization. Also, a TMER4 transcript retaining a stem-loop would be expected to interact with components of the RNA-induced silencing complex (RISC) machinery, independent of sequence specificity. A multitude of additional functions have been ascribed to TMER4, including acting as a pro-survival signal for the cell, blocking apoptosis of infected cells, suppressing an antiviral immune response, and initiating an immune response that is responsible for the movement of the infection into peripheral circulation. Each of these functions is essential for MHV-68 pathogenesis and the establishment of latency.

Another study established that one or all TMERs as essential molecular factors triggering the development of viral pneumonia in an immunocompromised host, as TMER-deficient MHV-68 showed reduced virulence, despite having an enhanced frequency of virus-infected cells [84]. Strikingly, the expression of a single viral tRNA-like molecule, in the absence of all other virus-encoded TMERs and miRNAs, reverses both attenuation in virulence and the enhanced frequency of infected cells. These data show that TMERs play essential functions in acute infection and virulence in immunocompromised hosts and identify them as a new target to modulate MHV-68 infection and pathogenesis.

## 6. Herpesvirus Saimiri-Encoded lncRNAs

**Figure 4:**
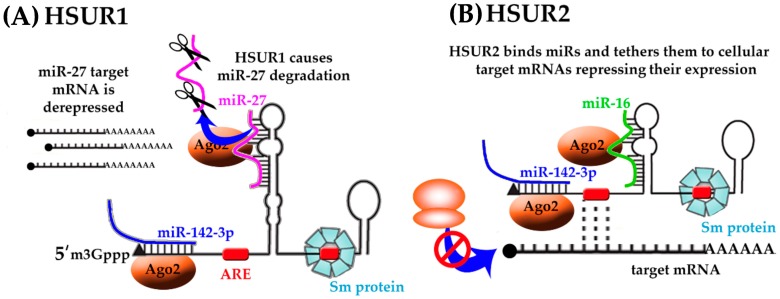
Putative functions of HSUR RNAs (**A**, HSUR1; **B**, HSUR2). HSURs have been found to have highly conserved regions responsible for binding of host miRNAs, i.e., miR-16 (green), miR-27 (pink), miR-142 (blue) and host proteins, i.e., Ago2 (orange), involved in RISC complex formation, spliceosomal Sm proteins (blue). While the exact mechanism and function of HSURs are not yet understood the recruitment of host miRNAs and proteins likely regulates gene expression of the target messenger RNA.

Herpesvirus saimiri (HVS) establishes latency in the T cells of New World primates and it has the ability to cause aggressive leukemias and lymphomas [43,85]. During latency HVS expresses seven small nuclear uracil-rich non-coding RNAs, called HSURs (Figure 4) [39,40,41,43]. The HSURs have common features with Sm-class small nuclear RNAs (snRNAs) and share the same biogenesis pathway. They are transcribed by RNA pol II in the nucleus, and subsequently exported to the cytoplasm, where they associate with Sm core proteins and acquire a trimethylguanosine 5′ -end cap before being imported back to the nucleus.

The 5′ termini of HSURs 1, 2, and 5 contain a highly conserved the AUUUA pentamer characteristic of AREs that regulate the stability of many host mRNAs [86]. In the case of HSUR1, the ARE-like elements associate with cellular proteins hnRNPD and HuR, to mediate ARE-dependent degradation pathway, leading to HSUR1 decay [87].

HSUR1 and HSUR2 have been shown to modulate the expression of transcription factors that are involved in the apoptotic response, cell cycle checkpoints, and cellular metabolism, i.e., fork-head box1, FOXO1, PAX3, and RUNX1 [88,89,90]. Cazalla et al. observed complementarity between HSUR1 and HSUR2 sequences and the seed regions of three host miRNAs, namely, miR-161, miR-27, and miR-42 (Figure 4). Both HSURs have been also found in Argonaute 2 (Ago2) complexes from HVS-transformed marmoset cells [42].

HSUR1 reduces miR-27 levels in infected marmoset T cells through target RNA-directed miRNA degradation (TDMD), thereby derepressing miR-27 cellular target mRNA production and promoting T cell activation (Figure 4A). TDMD of miR-27 is required for efficient HSV replication, as viral strains with HUSR1 bearing a mutated miR-27 binding site have reduced titers [91]. Knockdown of HSUR1 confirmed the negative effects of HSUR1 on miR-27 accumulation and mutation of the miR-27 complementary sequence in HSUR1 abolished the interaction and the reduction of miR-27 levels.

Conversely, HSUR2 does not deplete the miRNAs that it binds, but instead acts as a tether that recruits the Ago–miR-142-3p and Ago–miR-16 complexes to cellular mRNAs that encode pro-apoptosis factors. These complexes then induce the silencing of these tethered mRNAs and thus prevent apoptosis (Figure 4B) [92]. In addition, the in vivo crosslinking analysis indicated that, also, HSUR2 base-pairs with mRNAs encoding retinoblastoma and factors that are involved in p53 signaling and apoptosis.

## 7. Gammaherpesvirus-Encoded lncRNAs as Therapeutic Targets

As discussed above, gammaherpesvirus-encoded lncRNAs are key modulators of viral pathogenesis and replication. Thus, they represent an as of yet unexplored opportunity for pharmacological intervention as specific targets in the context of structure–function relationships. The currently available therapeutic options targeting gammaherpesviruses rely mostly on the application of nucleoside analogs that selectively inhibit lytic replication of the virus [93]. These antiviral strategies are limited by the continual emergence of resistant strains [94], and the fact that the latent viral reservoir is not eliminated.

RNA therapeutics can take advantage of the unusually high abundance of viral lncRNAs, the presence of these molecules at various stages of viral life cycle, often including latency, and the parts that are played in crucial functions in cellular processes and pathogenesis. For example, EBV-encoded EBERs have been suggested as potential therapeutic targets due to their indispensable roles in pathogenesis of nasopharyngeal carcinoma (NPC) [95,96,97] and lymphoproliferative diseases [98]. Another emerging therapeutic target are the EBV-related exosomes that can be used as biomarkers for EBV-associated cancers. These extracellular vesicles, which are shown to contain both EBERs [99], are also known to aggravate the progression and invasion of various cancers [100,101,102], and as such, they represent as of yet unexplored therapeutic target. It has been reported that the tumor-derived exosomes could be the major reasons for the treatment failure in cancers [103,104]. In this regard, cancer progression could be inhibited by blocking the lncRNAs that are associated with these exosomes. Frequently, a single lncRNA scaffolds or targets several molecular factors, therefore the manipulation of the levels or stability of this RNA may serve to modulate the functions of multiple viral genes. For example, PAN RNA is a key molecular switch that directs KSHV lytic reactivation by modulating the expression of numerous viral and host genes. PAN RNA has been also shown to be the most abundantly expressed viral transcript, not only in KSHV-lytically infected cells, but also in KSHV-associated tumors [14]. Targeting PAN can lead to the disruption of viral latency, and combing such approach with currently available antivirals, might be an effective strategy for eradication of KSHV infection and prevention of KSHV-associated malignancies. Although an EBV homologue of PAN RNA is unknown at this time, BHLF1, has been suggested to preform analogous to PAN RNA functions in regulating EBV lytic reactivation [14].

Numerous RNA-based therapeutic approaches for antiviral treatments have been developed, with each having its own impediments, as well as benefits [105,106,107]. siRNA- or antisense oligonucleotide (ASO)-mediated knockdown strategies that are designed to disrupt lncRNAs function are often cytotoxic, have poor intracellular uptake, and this application is frequently linked with off-target effects. In addition, single-stranded ASOs have often reduced stability, are frequently subjected to cellular degradation, and sometimes have low target affinity and potency. To overcome these issues, chemical modifications to ASOs, including the addition of 2′-*O*-methyl and/or locked nucleic acid (LNA) bases, have been shown to increase affinity, improve cellular uptake and decrease toxicity [108]. Off-target hybridization effects can also be minimized by cautious bioinformatic selection of ASO sequences, while the problems that are associated with systemic ASOs delivery are tackled by combining oligonucleotides with delivery enhancing agents [109]. siRNA or ASO-mediated strategies can be of particular application against gammaherpesvirus-encoded lncRNAs that are known to manifest their function by binding to chromatin-modifying complexes, i.e., PAN RNA [55]. In these cases, lncRNA docking onto the protein surface can be sequence- or structure-dependent. Uniformly modified ASOs that cannot stimulate RNAse H activity can be used in such cases to bind specifically to the lncRNA and block the RNA–protein interface, resulting in a loss of function.

Recently, small molecule targeting of RNA structures has emerged as a promising avenue against viral disease [105]. Small molecules offer the advantage of having desirable properties, such as good absorption, distribution, and oral bioavailability. They bind RNA by virtue of secondary or tertiary structure, as opposed to sequence, and as such, they provide an orthogonal means to target unique motifs [110,111]. Ligands binding to lncRNA architectures would be able to affect RNA–RNA, RNA–DNA, RNA–protein interactions, structural stability or conformational changes, and thereby block processes essential for viral replication. Proof-of-concept for targeting functional RNAs by small molecules has been demonstrated for multiple viruses, including HIV, HCV [111], SARS CoV [112], and Influenza [113].

Another challenge in developing an effective antiviral strategy against gammaherpesviruses is that lytic reactivation is needed before antiviral agents targeting virus can be employed. Most of the current therapeutics target viral products present only during a productive infection, and the latent virus reservoir is impervious to these treatments. Thus, latency represents an attractive target for viral eradication, and indeed, recent studies using latency-reversing agents showed effectiveness during the treatment of HIV infection [114,115,116]. Currently available latency-reversing agents against EBV and KSHV infections [115,117,118,119], manipulate an epigenetic pathway i.e., histone epigenetic modifications to achieve viral reactivation, and none of them target viral lncRNAs. In this review, we have deduced multiple examples of gammeherpesvirus-encoded lncRNA directly involved in viral lytic reactivation, i.e., KSHV PAN RNA, EBV BHLF1, and OriPts. Targeting them with therapeutics may lead to viral reactivation, which, in combination with other antiviral agents, can create a platform for effective eradication of gammaherpesvirus-associated infections. Also, recent developments in the field of host lncRNA deregulation by gammaherpesvirus latency-associated factors suggest that the therapeutic targeting of these interactions may also be feasible, especially since these contacts are latency specific. It has been shown that during KSHV latency, viral miRNAs and latency proteins target various host lncRNA implicated in cancer, including MEG3, ANRIL, TUG1, and UCA1 [120]. Also, EBV-associated miRNAs have been shown to target numerous host lncRNAs in a RISC-dependent manner [121]. Thus, studies on aberrantly expressed host lncRNAs in gammaherpesvirus-infected cells may aid the development of novel virus-specific therapeutic targets.

For each of the therapeutic approaches aiming at gammaherpesvirus-encoded lncRNAs, knowledge about the relationship between a drug’s systemic exposure (or concentration) and responses (beneficial and/or adverse) is key in selecting the most suitable antiviral therapy [122]. Other practical considerations, such as a copy number variation of target lncRNAs, must be considered. Some lncRNAs, despite being preferable targets, might simply not be readily expressed or functionally accessible at the desired stage of viral replication. Additionally, criteria of drugability, which we have discussed below, have to be thoroughly applied to select lncRNAs targets that are most suitable for the development of antiviral strategies.

## 8. Gammaherpesvirus-Encoded lncRNAs: Future Directions

**Figure 5:**
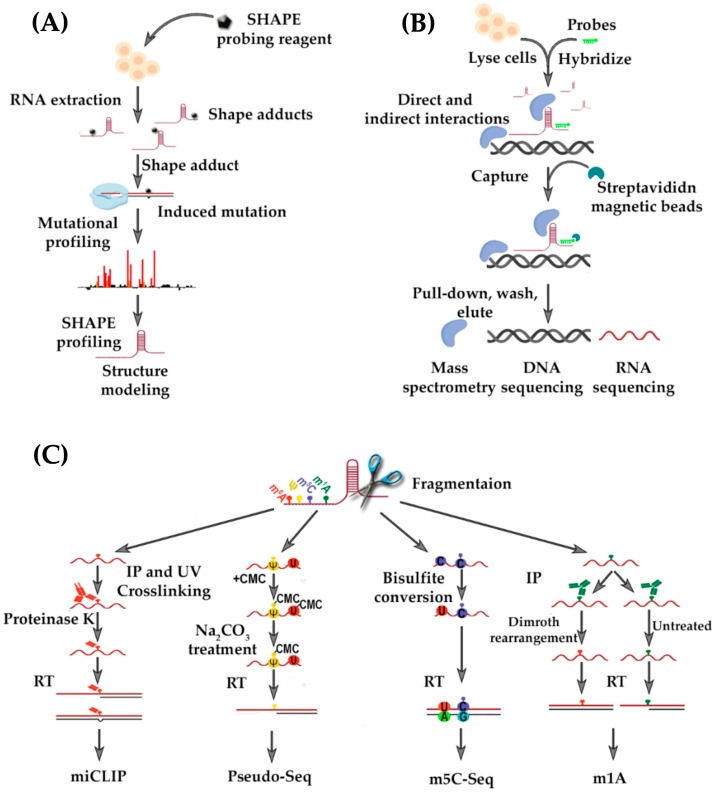
Novel molecular approaches to address the structure-to-function relationship of gammaherepsvirus-encoded lncRNAs. (**A**) RNA secondary structure analysis by selective 2′-hydroxyl acylation analyzed by primer extension (SHAPE) takes advantage of electrophilic chemical probes that target single-stranded or structurally unconstrained nucleotides and modify them at the 2′-hydroxyl group. When coupled with mutational profiling (MaP), the modified nucleotides are detected as internal miscoding nucleotides during reverse transcription followed by massively parallel sequencing; (**B**) RNA antisense purification (RAP) is used to purify a target lncRNA in complex with other RNAs, proteins and DNA. Biotinylated probes (light green) designed to associate with the target lncRNA (brown) are hybridized to the transcript that has been specifically cross-linked to interacting partners. Streptavidin magnetic beads (dark green) capture the biotinylated probes (light green) to pull-down the lncRNA and associated molecules, i.e., other RNAs, proteins, and DNA (blue) for further analysis by mass spectrometry, DNA-seq or RNA-seq; (**C**) Examples of deep-sequencing based mapping techniques that are used to address the four most common epitranscriptomic modifications in RNA. The *N*^6^-methyladenosine (m^6^A, red) is detected with individual-nucleotide-resolution cross-linking and immunoprecipitation (miCLIP) methodology. Here, immunoprecipitation (IP) and UV crosslinking with m^6^A-specific antibodies is coupled with reverse transcription and deep-sequencing, and the sites of modification are detected as either misincorporation of base-pairs or truncation. Pseudouridine (Ψ, yellow) is detected by CMC-derivatization, where sodium carbonate removes the CMC derivative from non-pseudouridine modifications. The 5-methylcytosine (m^5^C, purple) uses bisulfite conversion that causes non-methylated cytosines to be converted to guanine. The *N*^1^-methyladenosine (m^1^A, green), similar to miCLIP, relies on using m^1^A-specific antibodies.

Targeting gammaherpesvirus-encoded lncRNAs requires a comprehensive understanding of their structure to create an effective approach against viral infection and associated diseases. Structurally well-defined, ligand binding pockets are rare in RNA folds, but they provide the most promising opportunities for discovery of antiviral therapeutics. Targeted lncRNA must also be molecularly accessible and unobstructed by scaffolding contacts with other molecules that could potentially hamper the effective targeting. The application of deep-sequencing RNA structure probing techniques, including the selective 2′-hydroxyl acylation analyzed by primer extension and mutational profiling, (SHAPE-MaP [50]), as well as SHAPE-seq [123] and DMS treatment coupled with massively parallel sequencing readout (*DMS*-*seq*) [124], can offer an insight into the conformation of targeted lncRNAs, aiding the choice of a functional motifs against which one can successfully design a therapeutic approach (Figure 5A). Also, RNA-centric biochemical affinity techniques, such as RNA antisense purification (RAP) (Figure 5B) [125], chromatin isolation by RNA purification (ChIRP) [126], and capture hybridization analysis of RNA targets (CHART) [127] can be useful in revealing the comprehensive lncRNA interactome network, including contacts with chromatin, proteins, and other RNAs.

Another layer of RNA structure and function regulation has captured the attention of the scientific field, namely, epitranscriptomic modifications. Several of them, including *N*^6^-methyladenosine (m^6^A), 5-methylcytidine (m^5^C), inosine (I), pseudouridine (Ψ), and *N*^1^-methyladenosine (m^1^A), are present in lncRNAs and they influence metabolism, stability, structure, and function [128]. Advances in the development of high-throughput and site-specific sequencing technologies to identify distinct epitranscriptomic markers provide us with new tools to identify the location and dynamic distribution of RNA modifications, and to reveal how these modifications affect lncRNAs (Figure 5C). For example, lncRNA metastasis-associated lung adenocarcinoma transcript 1 (MALAT1) has been shown to contain m^5^C [129] and Ψ [130] modifications, however an influence on RNA structure-to-function relationship has yet to be proven. In addition, at least two m^6^A signatures have been localized to the MALAT1 stem-loop, which reduce its stability and facilitate the binding of heterogeneous nuclear ribonucleoprotein C [131]. While the *MALAT1* hairpin is the first example of so called m^6^A-switch, structural changes induced by the presence of m^6^A modification, this phenomenon likely applies to other lncRNAs. It would be interesting to address whether gammaherpesvirus-encoded lncRNAs also undergo epitranscriptomic modifications and how these changes might affect RNA structure and function during viral replication and pathogenesis. This information would contribute greatly to the unraveling of novel modes of action by lncRNAs, as well as revealing potential “weak spots” in the lncRNA-associated interactome that govern the gammaherpesvirus life cycle and pathogenesis.

## Figures and Tables

**Table 1:** Long non-coding RNAs (lncRNAs) in gammaherpesvirus. The table includes: aliases ascribed to the aforementioned lncRNAs, the molecular size expressed in kilobases, proteins associated with lncRNAs, the detection method and the original references.

Virus	Name	Alias(es)	Size (Kb)	Associated Proteins	Method of Detection	References
**KSHV**	K1/ORF4-11	ALE; K1.3; K1/11-AS	~17.0		RNA-seq, ribosomal profiling and tilling microarray	[9]
	vIL6/ORF2	K1.5	~1.0		Genome-tilling microarray	[26]
	vIL6/K4.2	K2/K4.2-AS	~5.8		RNA-seq and ribosomal profiling	[9]
	K4s	K3.5	~0.9		Tilling microarray	[27]
	PAN/K7	K7.3; anti-PAN	~1.3		Tilling microarray	[27]
	K9/ORF62		~17.3		RNA-seq and ribosomal profiling	[9]
	ORF58/59	K11.5	~2.5		Tilling microarray	[27]
	ORF65/69		~7.5		Tilling microarray	[27]
	vGPCR		~4.0		Tilling microarray	[27]
	ORF7		~0.8		Tilling microarray	[27]
	K5/K6	K4.5; T6.1; K5/6-AS	6.1		RNA-seq, ribosomal profiling	[9,28]
	IR	K4.7; T1.5	1.2		RNA-seq, ribosomal profiling	[9,28]
	PAN RNA	nut1; T1.1	1.1	hnRNP C1, PABPC1, LANA, ORF57, PCRC2	RNA-seq, ribosomal profiling	[9,28,29]
	RTA	K7.7; T3.0; 50L	2.9		RNA-seq	[9,28]
	RTA	T1.2; 50S	1		RNA-seq, ribosomal profiling	[9,28]
	miR/K13/72/LANA	ALT; K12.5	10.1; 9.9		Genome-tiling microarray	[26]
**EBV**	EBER1		0.167	La, L22, hnRNPD	RNA-seq	[30,31,32,33,34]
	EBER2		0.172	La, nucleolin, PAX5	RNA-seq	[30,31,34,35,36]
	EBV-sisRNA-2		2.971		Computational modeling and RNA-Seq	[37]
	BHLF1		2.5		Northern blot	[14,37]
	oriPtLs	Leftleftward oriPt	2.304	NONO, ADAR	Tilling microarray, RNA-seq, FISH	[11]
	oriPtRs	Rightward oirPt	2.304	NONO, ADAR	Tilling microarray, RNA-seq, FISH	[11]
**MHV-68**	TMER1		0.2–0.25	AGO2, Sm proteins	Plasmid construction and DNA	[38]
	TMER2		0.2–0.25	AGO2, Sm proteins	sequencing paired with tRNA search	[38]
	TMER3		0.2–0.25		programs, RT-PCR analysis	[38]
	TMER4		0.2–0.25			[38]
	TMER5		0.2–0.25			[38]
	TMER6		0.2–0.25			[38]
	TMER7		0.2–0.25			[38]
	TMER8		0.2–0.25			[38]
**HVS**	HSUR1		0.114–0.143	Sm, Ago2, HuR, hnRNP D	RNA-seq	[39,40,41,42]
	HSUR2		0.114–0.144	Sm, Ago2, HuR, hnRNP D	RNA-seq	[39,43]
	HSUR5		0.114–0.145	Sm, Ago2, HuR, hnRNP D	RNA-seq	[39,43]
	HSUR3		0.075–0.106	Sm	RNA-seq	[39,43]
	HSUR4		0.075–0.106	Sm	RNA-seq	[39,43]
	HSUR6		0.075–0.106	Sm	RNA-seq	[39,43]
	HSUR7		0.075–0.106	Sm	RNA-seq	[39,43]

Kaposi’s sarcoma-associated herpesvirus: KSHV; Herpesvirus saimiri: HVS; murine herpesvirus-68: MHV-68; Epstein-Barr virus: EBV; open reading frame: ORF; polyadenylated nuclear: PAN; replication and transcription activator: RTA; latency-associated nuclear antigen: LANA; Epstein-Barr virus-encoded RNA: EBER.
